# CD38 Drives Progress of Osteoarthritis by Affecting Cartilage Homeostasis

**DOI:** 10.1111/os.13258

**Published:** 2022-04-20

**Authors:** Jin‐jin Ma, Jun Ying, Jin‐yu Wang, Tao‐tao Xu, Han‐ting Xia, Hong‐ting Jin, Lu‐wei Xiao, Wen‐jun Shang, Wei‐qian Wang, Jian‐yin Feng

**Affiliations:** ^1^ School of Stomatology Zhejiang Chinese Medical University Hangzhou China; ^2^ Department of Orthopaedic Surgery The First Affiliated Hospital of Zhejiang Chinese Medical University Hangzhou China; ^3^ The Affliated Hospital of Jiangxi University of Traditional Chinese Medicine Nanchang China; ^4^ Hangzhou Stomatological Hospital Hangzhou China

**Keywords:** Cartilage, CD38, Chondrocyte, NAD, Osteoarthritis

## Abstract

**Objective:**

To observe expression of CD38, a key modulator of nicotinamide dinucleotide (NAD+) metabolism in mice with knee osteoarthritis, and protective effect of CD38 inhibition during the osteoarthritis (OA) development.

**Method:**

The destabilization of the medial meniscus (DMM) model was performed in mice to mimic the process of OA. Immunofluorescence of CD38 was performed to evaluate its response during the OA process. Limb bud‐derived mesenchymal cells were isolated for micromass culture. 100 nM or 1 μM CD38 inhibitor (78c) treatment for 14 days and CD38 sgRNA infection were then used to explore the effects of chondrogenic differentiation *via* Alcian blue staining. The expressions of chondrogenic markers were detected using RT‐PCR and Western blot. To explore the protective effect of CD38 inhibitor on cartilage degradation during OA *in vivo*, a CD38 inhibitor was injected into the knee joint after DMM operations. Micro‐CT analysis and Safranin O‐fast green staining were used to evaluate subchondral bone micro‐architecture changes and cartilage degeneration.

**Results:**

Compared to the control group, the CD38 expression in superficial cartilage was obviously increased in DMM group (*P* < 0.05). During the normal chondrogenic differentiation, the extracellular matrix formed and expression of *Sox9*, *Col2*, *aggrecan* increased apparently while CD38 expression decreased, which could be reversed with ablation of CD38 in limb bud‐derived mesenchymal cells. Consistent with findings *in vitro*, CD38 blockage *via* CD38 inhibitor injection protected against osteosclerosis in medial subchondral bone and cartilage degeneration in DMM‐induced experimental mice. Compared to the Sham group, DMM mice showed significantly increased values of BV and BV/TV in subchondral bone (*P* < 0.05) and Mankin score, which could be rescued by 78c treatment (*P* < 0.05). Also the CD38 inhibitor contributed to homeostasis of anabolism and catabolism by upregulating *Sox9*, *Col2*, *aggrecan* and downregulating *Runx2*, *Col10* and *Mmp13*.

**Conclusion:**

This study primarily implicates CD38 as an important regulator of chondrogenic differentiation. Inhibition of CD38 demonstrated protection against cartilage degeneration, which suggests that CD38 could be a potential therapeutic target for OA.

## Graphical abstract

CD38 is a membrane‐bound protein. This study primarily implicates CD38 as an important regulator of chondrogenic differentiation. Inhibition of CD38 demonstrated protection against cartilage degeneration, which suggests that CD38 could be a potential therapeutic target for OA.

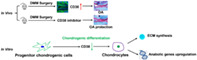

## Introduction

Osteoarthritis (OA) is a common age‐related disease, but its mechanisms remain poorly understood^1^. Human articular cartilage has been defined to four zones based on histological features: the superficial zone, formed of flattened chondrocytes; the middle zone, formed of small round chondrocytes; the deep zone, including larger round chondrocytes; and the mineralized articular cartilage. During OA initiation and progression the articular cartilage, subchondral bone, synovial tissue, and meniscus are all affected^2^. Because of load distribution and shock absorption and properties, meniscus is critical for maintaining the health of knee joint. Clinically, meniscal damage is often observed in the knees of patients with OA. Meniscal damage is considered to induce OA, while OA also results in meniscal damage^3^.

Over last decade, it has been demonstrated that NAD levels decrease with age and chronic diseases which result in metabolic abnormalities^4^, ^5^, ^6^. Reduced NAD levels in these circumstances may be caused by an increase in NAD‐consuming enzymes, including sirtuins, cyclic ADP‐ribose hydrolase (CD38‐ying, 2006) and poly (ADP‐ribose) polymerases (PARPs). Studies have reported that many NAD‐dependent protein deacetylases are closely related to OA lesions and play important roles in maintaining articular cartilage homeostasis^7^, ^8^, ^9^. Sirtuins (SIRT1–7) are involved in regulating a series of biological functions, including cartilage homeostasis, cartilage diseases and OA development. Wataru *et al*. has shown that knockdown of SIRT7 in the murine chondrogenic cells elevated the deposition of a glycosaminoglycan‐rich extracellular matrix and the mRNA expression of extracellular matrix components. Also, deletion of SITR7 in mice attenuated the development of aging‐related OA and forced exercise‐induced OA^10^. SIRT1 is also reported to play a key role in the OA pathological progress *via* the regulation of expression of extracellular matrix‐related proteins and mesenchymal stem cell differentiation. Phytochemical resveratrol shown the ability to suppress OA disease progression through activation of SIRT1^11^, ^12^. PARP1 is related to inflammatory response. Study demonstrated that inhibition of PARP1 suppresses interleukin 1b‐induced inflammation in human osteoarthritic chondrocytes, suggesting PARP1 is a potential therapeutic target of OA^13^.

CD38 (Cluster of differentiation 38) is a multifunctional protein that plays a role both as a cell surface expressed receptor and as an enzyme. CD38 is a membrane‐bound protein, first identified as a surface marker in lymphocytes^9^. Due to further research, CD38 expression is now considered ubiquitous across all tissues^7^. CD38 is found on the surface of many immune populations, including CD4+ cells, CD8+ cells, B lymphocytes and natural killer cells. As a receptor, CD38 can bind with CD31 which is located on the surface of T cells, thus resulting in the activation of those cells and production of a variety of cytokines^14^. Recent studies have also identified CD38 as a key modulator of nicotinamide dinucleotide (NAD+) metabolism, its first functional implication as an enzyme. The main enzymatic activity of CD38 is through the hydrolysis of NAD to nitocinamide and ADPR. Additionally, CD38 catalyzes NAD+ to cylic ADP‐ribose (cADPr), a Ca^2+^ releasing second messenger.CD38 protein is a marker of cell activation and associated with a variety of biological functions and diseases, including leukemias, myelomas and diabetes. CD38 is suggested as a suitable target for adult leukemia^15^. The activation of CD38 is considered as an alternative signaling pathway for glucose‐stimulated insulin release in human beta‐cells^16^. CD38 also plays a critical role in bone remodeling, mainly in the regulation of osteoclast formation and bone resorption^17^. Besides, NAD metabolism and its dysfunction *via* the enzyme CD38 are involved in the pathogenesis of rheumatologic diseases, including systemic sclerosis, systemic lupus erythematosus and rheumatoid arthritis. The inhibition of CD38 enzymatic activity might be a promising therapeutic target^18^.

Since the emerging role of NAD and NAD‐regulating enzymes, like sirtuins and PAPR, in the pathogenic process of OA, we hypothesized that: (i) CD38 is involved in the OA progression; (ii) CD38 plays an important role in articular cartilage homeostasis of anabolism and catabolism; and (iii) regulating CD38 abnormality can prevent cartilage degeneration and OA development. In this study, we focus on the role of CD38‐mediated OA development. Our results suggest that CD38 inhibitor treatment not only prevents articular cartilage degradation, but also subchondral bone sclerosis, opening up the possibility of using CD38 to protect against OA development.

## Materials and Methods

### Animals and Groups

All animal studies were performed in accordance with approval of the Animal Care and Use Committee in Zhejiang Chinese Medical University. 10‐week‐old male C57BL/6 mice were purchased from the animal center of Zhejiang Chinese Medical University (Y2111184). Mice were anesthetized with intraperitoneal injection of ketamine (80 mg/kg). Destabilization of the medial meniscus (DMM) surgery was performed by transecting medial meniscotibial ligament (MMTL) in 10 weeks old C57BL/6 male mice, which were considered the animal model of osteoarthritis. The sham group only underwent skin and joint capsule incisions.

All mice were randomly divided into four groups: Sham group (in which only the capsule of the knee joint was cut), CD38 inhibitor (78c) group (capsule cut+78c local injection), DMM group (MMTL cut) and DMM + 78c group (MMTL cut+78c local injection). CD38 specific inhibitor, 78c was intra‐articularly injected at 6 μg dissolved in 10μl 0.1% dimethyl sulfoxide(DMSO) per knee joint four times a week. The mice were sacrificed for sample collection 6 weeks post‐operation.

### Reagents and Antibodies

Reagents used were as follows: 78c (S8960, Selleck, Radnor, PA, USA); Ethylenediaminetetraacetic acid (EDTA) (17,892, Thermo Fisher Scientific, Waltham, PA, USA); bovine serum albumin (BSA) (9048‐46‐8, Sigma‐Aldrich, St. Louis, MO, USA); Trypsin (15,400,054, Invitrogen, Carlsbad, CA, USA); Recombinant Bone morphogenetic protein 2 (BMP2) (78,004, Stemcell, San Antonio, TX, USA); Dulbecco's Modified Eagle Medium (DMEM) (11,885,084, Gibco, Big Cabin, OK, USA); FBS (26,140,079, Life Technologies, Frederick, MD, USA); Cas9 enzyme (LentiCRISPR v2; Addgene plasmid repository #52961, Watertown, MA, USA); Alcian blue solution (B8438, Sigma‐Aldrich, USA); Safranin O (477–73‐6, Sigma‐Aldrich, USA); Fast Green (2353‐45‐9, Sigma‐Aldrich, St. Louis, MO, USA); Primary antibodies used were as follows: anti‐CD38 antibody (sc‐37,465, Santa Cruz, USA); anti‐β‐actin (A1978, Sigma‐Aldrich, St. Louis, MO, USA), anti‐Col2 (SAB4500366‐100UG, Sigma‐Aldrich, USA) and anti‐aggrecan (AB1031, Sigma‐Aldrich, St. Louis, MO, USA).Secondary antibodies used were as follows: Goat anti‐rabbit immunoglobulin‐G (ab150077, Abcam, Waltham, PA, USA); Fluorescent secondary antibody (926–32,212, LI‐COR, USA). 4′,6‐diamidino‐2‐phenylindole (DAPI) (P36931, Invitrogen, Carlsbad, CA, USA) was used to stain *nucleus*.

### Micro‐CT Analysis

Samples were scanned at 10‐micron isotropic resolution using amicro‐computed tomography (Micro‐CT) (Skyscan 1176; Bruker μCT, Kontich, Belgium). Bone volume and bone volume fraction (BV/TV) were measured using the Skyscan analysis software. The region of interest(ROI) was focused on the medial tibial plateau.

### Histology and Immunofluorescence Analyses

Samples were fixed in 10% normal buffered formalin for 3 days, then decalcified with 14% EDTA for 10 days. Subsequently, decalcified samples were processed, embedded in paraffin, and sectioned at a thickness of 5 μm. Sections were deparaffinized, rehydrated, and stained with safranin‐O/fast green solution. Osteoarthritis Research Society International (OARSI) modified Mankin scoring criteria was used to evaluate cartilage degeneration by three blinded reviewers^8^.

To explore the CD38 expression in chondrocytes *in vivo*, immunofluorescence (IF) analysis was also performed on sections. After blocking unspecific binding with 3% BSA‐supplemented TBS for 1 hour, sections were incubated with anti‐CD38 (1:200), anti‐Col2 (1:1000) and anti‐MMP13 (1:100) antibodies overnight at 4°C. Sections were stained with DAB chromogen.CD38 antibody incubated sections were then covered with goat anti‐rabbit immunoglobulin‐G (1:2000) and 4′, 6‐diamidino‐2‐phenylindole (DAPI) (1:2000) to produce measurable signal. GFP fluorescence was imaged on Confocal Microscope (Zeiss LSM 880 II, Germany).Thefluorescence density was analysied by usingImage J Software.

### Limb Bud–Derived Mesenchymal Cell Isolation and Micromass Culture

Limb buds from E11.5 embryos were dissected and digested with Trypsin at 37°C for 20 mins^19^. Cell suspensions were then filtered through a 40‐μm cell strainer and centrifuged to collect the cells. The final density of cells was 1× 10^7^/ml. 10 μl cell suspensions were seeded in 24‐well plates for 2 hours, then cultured in DMEM supplemented with 10% FBS. Cells were divided in to three groups: Ctrl group (only treated with 100ng/mL BMP2), 78c‐100nM group (100ng/mL BMP2 + 100nM 78c treatment) and 78c‐1μM group (100 ng/mL BMP2 + 1 μM 78c treatment). After 14 days, alcian blue staining was performed on micromasses to evaluate the role of CD38 in chondrogenesis of limb bud‐derived mesenchymal cells. Briefly, the micromass was fixed with 10% neutral buffered formalin (NBF) for 30 mins. 1% Alcian blue solution was then used to stain for 30 mins before washing three times with 1 × PBS.

### Real‐Time Polymerase Chain Reaction Analysis

RNA was isolated from primary micromass cells and cartilage samples from mice using the RNeasy Mini Kit (QIAGEN). NanoDrop 2000 was used to measure RNA quality and concentration. Complementary DNA (cDNA) was synthesized using iScripts cDNA Synthesis Kit (Bio‐Rad, Hercules, CA, USA). Real‐time polymerase chain reaction (RT‐PCR) was performed using murine gene specific primers and the SYBR green real‐time PCR kit (Bio‐Rad, Hercules, CA, USA). Primer sequences for CD38, Sox9, Col2a1, aggrecan and β‐actin are as shown in Table 1.

**TABLE 1:** Primers and sequences for RT‐PCR analysis

Primers	Sequences
*β‐Actin* forward	5′‐CGTCCCGTAGACAAAATGGT‐3′
*β‐Actin* reverse	5′‐TTGATGGCAACAATCTCCAC‐3′
*CD38* forward	5′‐TTGCAAGGGTTCTTGGAAAC‐3′
*CD38* reverse	5′‐CGCTGCCTCATCTACACTCA‐3′
*Sox9* forward	5′‐CGGCTCCAGCAAGAACAA‐3′
*Sox9* reverse	5′‐TGCGCCCACACCATG‐3′
*Col2a1* forward	5′‐CTACGGTGTCAGGGCCAG‐3′
*Col2a1* reverse	5′‐GTGTCACACACACAGATGCG‐3′
*aggrecan* forward	5′‐GGAGCGAGTCCAACTCTTCA‐3′
*aggrecan* reverse	5′‐CGCTCAGTGAGTTGTCATGG‐3′
*Col10a1* forward	5′‐ATGCCTTGTTCTCCTCTTACTG‐3′
*Col10a1* reverse	5′‐TGCTGAACGGTACCAAACG‐3′
*Mmp13* forward	5′‐AGACTGGTAATGGCATCAAGG‐3′
*Mmp13* reverse	5′‐GCCATTTCATGCTTCCTGATG‐3′
*Runx2* forward	5′‐CGTCCACTGTCACTT TAATAGCTC‐3′
*Runx2* reverse	5′‐GTAGCCAGGTTCAACGATCTG‐3′

### Western Blot Analysis

Proteins were extracted from primary micromass on day 5 in culture using radio‐immunoprecipitation assay (RIPA) lysis buffer, containing 1 mM phenylmethylsulfonyl fluoride (PMSF) and a protease inhibitor cocktail. A BCA Protein Assay kit (Thermo Scientific) was used to detect protein concentration. 50 μg of protein was loaded and electrophoresed on mini protean gels (Bio‐Rad Laboratories, Hercules, CA, USA) and transferred to Immun‐Blot PVD Membranes (Bio‐Rad Laboratories). The membrane was then blocked in 3% BSA in TBST and incubated in primary antibodies, including β‐actin (1:1000), Col2 (1:500) and aggrecan (1:500). Membranes were incubated with a fluorescent secondary antibody (1:1000) for 1hr before detecting chemiluminescence using LI‐COR Odyssey® scanner (LI‐COR Biosciences, Lincoln, NE, USA) and software.

To slice CD38 in primary micromass cells using CRISPR‐Cas9, CD38 sgRNA were designed to be expressed from a U6 promoter. Cells were infected with a lentivirus carrying a plasmid including the Cas9 enzyme and an sgRNA against CD38. A nonsense scrambled sgRNA was also transduced into cells as a negative control. Primary micromass cells were infected with the lentivirus for 24 hours, then returned to normal medium.

### Statistical Analysis

Statistical analyses were performed using GraphPad Prism. All data are presented as the mean ± SD. Two‐way ANOVA followed by the Tukey test and unpaired Student's *t*‐test were used for statistical analyses. *P*‐value <0.05 was considered significant.

## Results

### CD38 Expression Associated with OA Progression

**Fig. 1:**
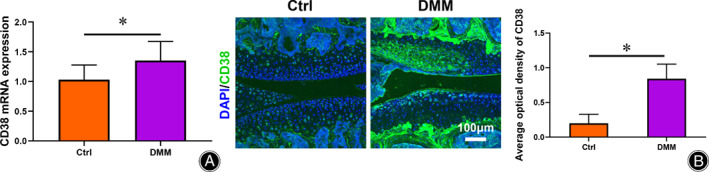
CD38 mRNA (A) and protein expression (B) were detected by RT‐PCR and immunofluorescence in control and DMM groups. CD38 expression was upregulated during the development of osteoarthritis. Quantitative analysis of the relative density of CD38 by densitometric analysis. Scale bar = 100 μm; Data are presented as mean ± SD. *P < 0.05, *n* = 6.

To ascertain whether CD38 plays a role in OA pathogenesis, we first analyzed the expression patterns of CD38 in mice with Sham and DMM surgery at 2 weeks post‐operation *via* immunostaining of knee joint sections(Fig. 1A and B). CD38 was broadly expressed in the superficial zone, middle zone and deep zone of both groups (Fig. 1B). However, DMM surgery induced a substantial increase of CD38 expression in superficial cartilage. The change in CD38 expression in mice with experimental OA indicates the potential important role of CD38 in articular cartilage degradation.

### Expression of CD38 Decreases during Chondrogenic Differentiation

**Fig. 2:**
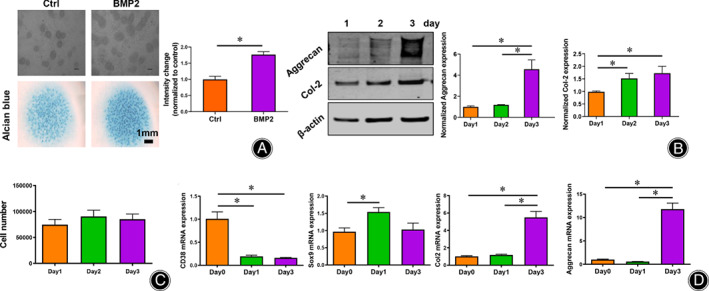
CD38 and chondrogenic marker expression during differentiation. (A) Cartilaginous nodules and extracellular matrix formed after 6 days of chondrogenic differentiation (*n* = 3). Alcian blue staining intensity was measured using Image J software. Scale bar = 1 mm; (B) Western blot was performed to detect the protein level of Col‐2 and Aggrecan in progenitor chondrogenic cells (*n* = 3). Quantification of the protein expressions was obtained using Image J software. (C) There is no obvious changes in cell quantity during the first three days of cell differentiation (*n* = 6). (D) CD38 gene expression decreased and Sox9, Aggrecan and Col2 increased during chondrogenic differentiation (*n* = 3). Data are presented as mean ± SD. *P < 0.05.

To elucidate the role of CD38 during chondrogenic differentiation and investigate how CD38 influence this process, we performed micromass culture. In this well‐established cell culture system, progenitor chondrogenic cells are harvested from embryonic limb buds and seeded at high cell density in spot cultures in which they keep their differentiation program and form cartilaginous nodules. After 6 days of differentiation, we stained the cells with Alcian Blue which showed positive cartilage nodules (Fig. 2A). We then performed Western blot and RT‐PCR to confirm chondrogenic expression. Consistently, all tested markers including *Sox9*, *aggrecan* and *Col2* increased during differentiation. However, CD38 expression was down regulated during chondrogenic differentiation, suggesting that the CD38 may act as a regulator in the articular cartilage, while no obvious changes of cell quantity was observed during the first three days(Fig. 2B–D).

### Inhibition of CD38 Activity Accelerates Chondrogenic Differentiation

**Fig. 3:**
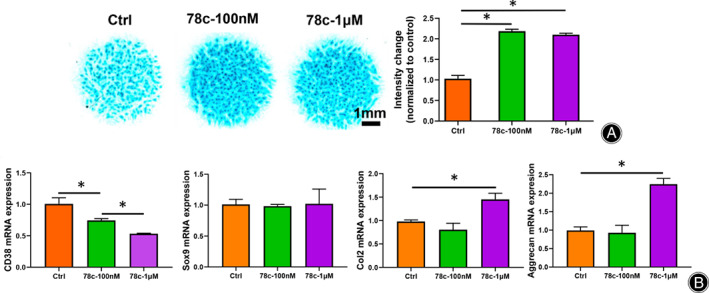
Ablation of CD38 promote chondrogenic differentiation. (A) 100 nM and 1 μM 78c treatment increases extracellular matrix formation in micromass cultures (*n* = 3). Alcian blue staining intensity was measured. Scale bar = 1 mm; (B) Compared with 100 nM 78c treatment, 1 μM significantly inhibit *CD38*mRNA expression and upregulate *Col2* and *aggrecan* mRNA expression (*P* < 0.05), but has no effect on *Sox9* mRNA expression (*n* = 3). Data are presented as mean ± SD. * *P* < 0.05.

Given our results showing decreased CD38 expression during chondrogenesis, we then considered whether inhibition of CD38 would have positive effect on chondrogenesis. To explore this possibility, we used a highly specific thiazoloquin(az)olin(on)es CD38 inhibitor, 78c, to pharmacologically inhibit CD38 *in vitro*. Based on previous work, we chose two concentrations with which to treat micromass cells, 100 nM and 1 μM. We found that 78c treatment strongly stimulated the formation of Alcian Blue‐positive chondrogenic nodules (Fig. 3A). Whole cell RNA samples were then extracted for RT‐PCR analysis. 78c could significantly inhibit *CD38* mRNA expression in a dose‐dependent manner (*P* < 0.05). Consistently, the data showed that 1μM 78c significantly increased the *Col2* and *aggrecan* mRNA expressions, but had no obvious effect on *Sox9* mRNA expression(Fig. 3B).

### CRISPR/Cas9 Lentivirus Knockout of CD38 Accelerates Chondrogenic Differentiation

**Fig. 4:**
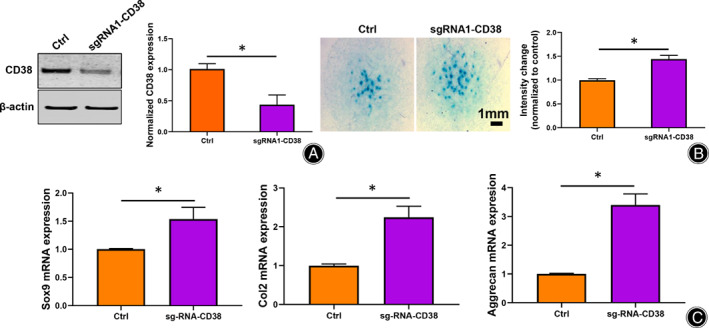
CRISPR/Cas9 Lentivirus knockout of CD38 promote chondrogenic differentiation. A. CRISPR/Cas9 Lentivirus transfection effectively decreased CD38 expression in primary micromass cells (*n* = 3). Quantification of CD38 protein expression was analyzed. B. Alcian‐Blue positive nodules are increased with CD38 knockout (*n* = 3). Scale bar = 1 mm; C. CD38 knockout significantly increased Sox9, Col2 and aggrecan gene expression (*n* = 3). Data are presented as mean ± SD. * *P* < 0.05

To fully understand the role of CD38 in chondrogenesis, we conducted CRISPR/CAS9 knock out of CD38 in the primary micromass cells. A plasmid expressing non‐targeting guide RNA was used as a parallel control to CD38. 293T cells were used to package control and CD38 knockout lentiviruses. Efficiency of CD38 knockout in primary micromass cells was verified using western blot (Fig. 4A). We next examined whether CD38 knockout affected chondrogenesis. Compared to the control group, CD38 knockout increased the number of Alcian‐Blue positive nodules (Fig. 4B). To further confirm the effects of CD38 knockout on chondrogenesis, RT‐PCR was performed to determine chondrocyte marker expression levels. Upon knockout of CD38, levels of Sox9, Col2 and Aggrecan increased (Fig. 4C).

CRISPR/Cas9 Lentivirus knockout of CD38 promote chondrogenic differentiation. (A) CRISPR/Cas9 Lentivirus transfection effectively decreased CD38 expression in primary micromass cells (*n* = 3). Quantification of CD38 protein expression was analyzed. (B) Alcian‐Blue positive nodules are increased with CD38 knockout (*n* = 3). Scale bar = 1 mm; (C) CD38 knockout significantly increased *Sox9*, *Col2* and *aggrecan* gene expression (*n* = 3). Data are presented as mean ± SD. * *P* < 0.05.

### CD38 Inhibitor Attenuates Articular Cartilage Degradation in Mice with Experimental OA

**Fig. 5:**
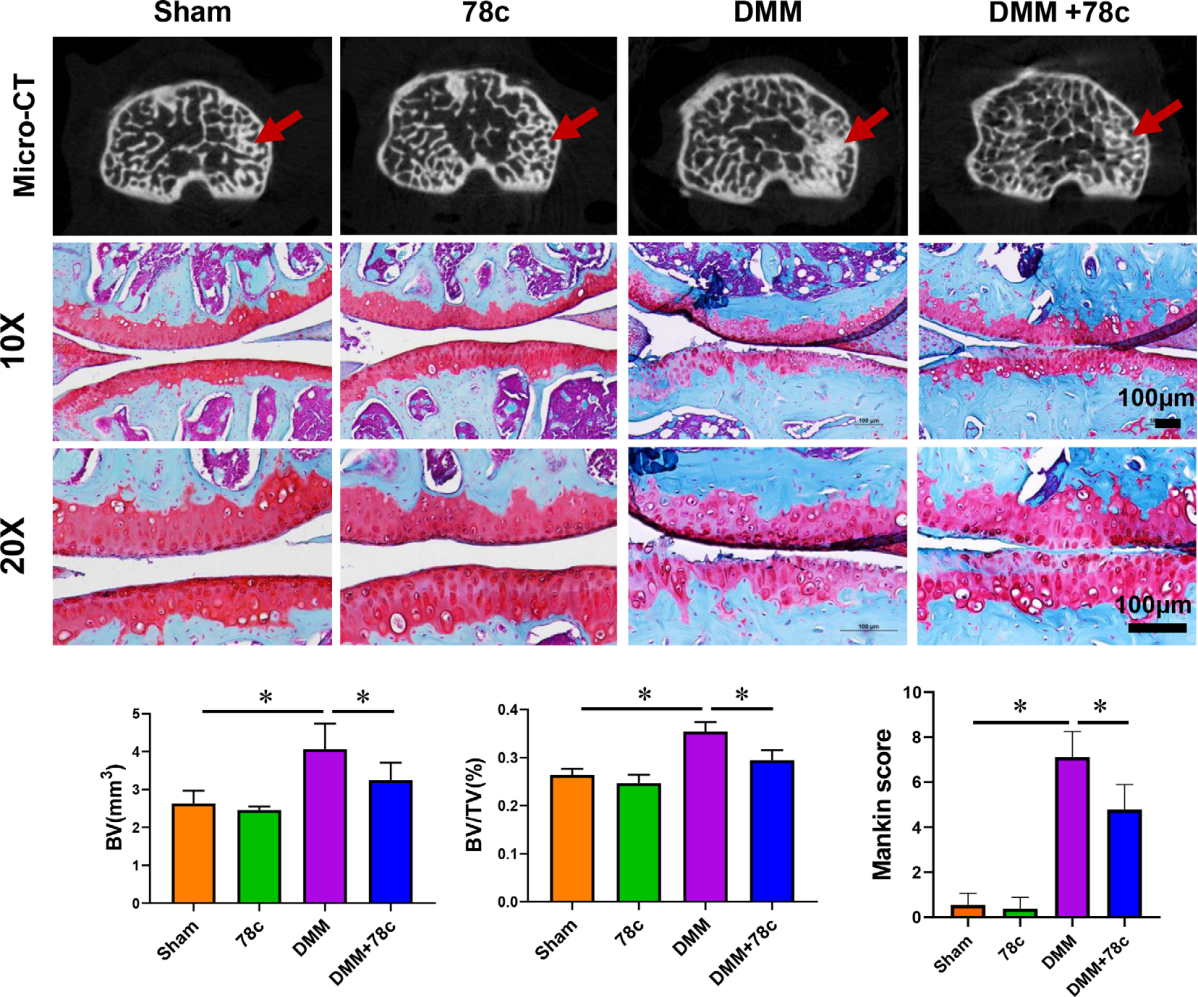
78c protected against cartilage degeneration in DMM‐induced mice. Micro‐CT analysis andSafranin O‐fast green staining were performed to evaluate the degree of joint degeneration. Scale bar = 100 μm; Data are presented as mean ± SD. * *P* < 0.05, *n* = 12.

Next, we investigated the potential role of CD38 in mice with experimental OA by intra‐articular injection of CD38 inhibitor(78c) into mice after sham or DMM operations. As shown in Fig. 5, DMM operation promoted osteosclerosis in medial subchondral bone, the effects of which were rescued by 78c administration. Compared to the Sham group, BV and BV/TV were significantly increased in the DMM group (*P* < 0.05). 6 weeks after treatment with the 78c, BV and BV/TV were significantly decreased (*P* < 0.05).

The articular cartilage of DMM mice showed an obvious loss of cartilage integrity, including loss of Safranin O staining. As expected, 78c treatment partially prevented cartilage degradation in DMM group mice when compared with DMSO injected controls. In agreement with the histological changes, modified Mankin scoring demonstrated that 78c apparently rescued histological scores in DMM mice at 6 weeks post‐surgery (*P* < 0.05).

### Disrupted Balance between Anabolism and Catabolism Is Rescued by CD38 Inhibitor in OA Mice

**Fig. 6:**
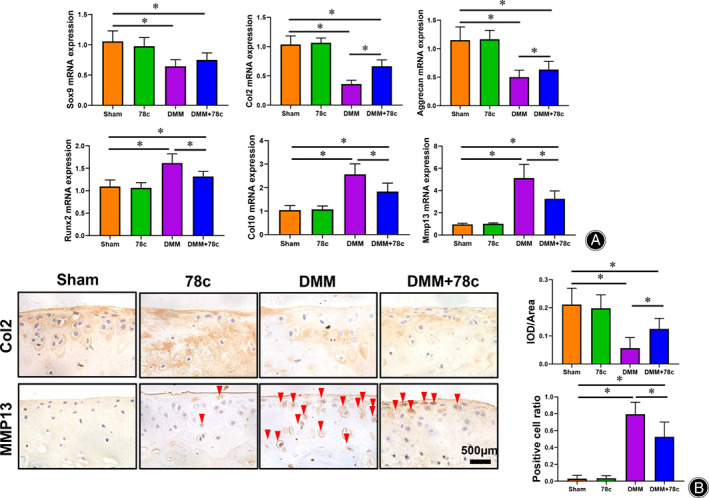
(A) 78c stimulates upregulation of anabolic genes *Sox9*, *Col2* and *aggrecan*, and downregulates hypertrophic gene *Col10* and catabolic markers *Runx2* and *Mmp13* in DMM‐induced mice (*P* < 0.05). (B) Col2 and MMP13 protein expression were assessed by immunohistochemistry and its quantitative analysis. Red arrows: MMP13‐positive cells. Scale bar = 500 μm. Data are presented as mean ± SD. * *P* < 0.05, *n* = 6.

To determine the changes to anabolism and catabolism in cartilage, genes of *Sox9*, *Col2*, *aggrecan*, *Col10*, *Runx2* and *Mmp13* levels were measured using RT‐PCR(Fig. 6A). Compared to the Sham group, anabolic genes including *Sox9*, *Col2* and *aggrecan* were significantly decreased *in the DMM group*, while hypertrophic marker *Col10* and catabolic markers *Runx2* and *Mmp13* were significantly increased (*P* < 0.05). Interestingly, with CD38 inhibitor (78c) treatment after 6 weeks, Col2 and aggrecan expression was increased, while *Col10*, *Runx2*, *Mmp13* were inhibited significantly relative to DMM group with no 78c treatment (*P* < 0.05). IHC staining for Col2 and MMP13 showed same changes as gene expression in each group (Fig. 6B). The changes in expression of anabolic and catabolic genes implicate CD38 as an effective target for altered homeostasis of cartilage matrix in the treatment of osteoarthritis.

## Discussion

Osteoarthritis (OA) is a common degenerative osteoarthropathy, characterized by progressive extracellular matrix (ECM) destruction, articular cartilage degeneration, and subchondral bone abnormality^20^, ^21^. Previous studies have demonstrated that abnormal homeostasis of chondrocytes, including apoptosis and unbalanced anabolism and catabolism, contributes to the OA development^22^.

### The Change of CD38inchondrocytesis Associated with OA Development

CD38 has been proven to be correlated with autoimmune diseases like Systemic Lupus Erythematosus (SLE), systemic sclerosis (SSc) and rheumatoid arthritis (RA)^23^, ^24^, ^25^. However, whether or not CD38 is involved in the progression of OA is still unclear. In this study, we firstly demonstrated that CD38 expression level increased in experimental OA joints. The experimental OA mouse model provided a highly reproducible progressive OA disease model^26^. Increased CD38 level of cartilage was observed in the DMM group. The current results provide an insight into how CD38 may act as a metabolic sensor^27^.

### CD38 Regulated Cartilage Homeostasis of Anabolism and Catabolism

CD38 is broadly expressed in inflammatory cells, and OA development is associated with inflammation processes^28^, ^29^, ^30^, ^31^. Inflammatory cytokines and other inflammatory mediators are generated by synovium and chondrocytes, and can be detected in the synovial fluid of OA patients. However, the mechanism by which the inflammatory process begins in OA is uncertain. Our results show increased CD38 expression in DMM induced experimental OA mice. It is possible that inflammation arising during OA may lead to an increase in the expression of CD38 in the joint, including the entire synovial complex. Although our studies demonstrated a protective effect of CD38 inhibitor in experimental OA, further work is necessary to elucidate the specific mechanisms. Subsequently, we provided *in vitro* data showing inhibition of CD38 in primary micromass cells, upregulated chondrogenic markers including Col2 and aggrecan. Furthermore, genetic knockout studies of CD38 indicated that the expression level of CD38 is important for maintaining normal chondrogenesis.

Inflammatory mediators lead to a vast array of downstream signaling pathways, including NF‐κB and MAPK pathways in the articular chondrocytes. Studies have demonstrated that CD38 expression is induced by the inflammatory cytokine TNF‐α in the human airway smooth muscle cells, leading to increased intracellular calcium response^32^. MAPK and NF‐κB pathways are involved in this process by regulation of CD38 expression.

### Ablation of CD38 Prevent Cartilage Degeneration

One of the most important risk factors for OA is age. A high percentage of people over the age of 65 have joints changes, including effects on cartilage, synovium, subchondral bone and muscle^33^, ^34^. Previous studies have demonstrated that expression and activity of CD38 increase with aging. In this study, the results clearly showed that CD38 protein and mRNA levels increased in several tissues, leading to reduced NAD levels in the tissues^35^. It would thus be interesting to look at the CD38 expression in the cartilage of aging mice. This and other recent studies highlight the potential therapeutic role of specific CD38 inhibitors to treat NAD related diseases^36^.

CD38 was one of the main enzymes which associated with age‐related NAD decline in mammals, and that CD38 knockout mice were protected against this progressive deficit^35^. Our study used a CD38 inhibitor, 78c, to treat experimental OA mice for 6 weeks. Interestingly, 78c treatment apparently attenuated the development of OA by reducing articular cartilage degradation and subchondral bone sclerosis.

## Conclusion

CD38 is associated with articular cartilage degeneration. We present strong evidence that CD38 is an essential enzyme involved in DMM‐induced OA development. In addition, CD38 inhibition facilitated extracellular matrix accumulation, as well as preventing cartilage damage. Regulating the CD38 expression may be a therapeutic approach to protect the cartilage against osteoarthritis.

## Conflict of Interest

The authors have declared that there is no conflict of interests in this work.

## Author Contributions

W.W. and J.F. designed the experiments; J.M., J.Y., J.W. and H.X. completed the experiments; T.X., H.J. and L.X. analyzed the experimental data; W.W. and J.M. edited the manuscript. J.F. reviewed the manuscript. All authors approved the final submitted manuscript.
